# United States Pharmacopœia

**Published:** 1877-03

**Authors:** 


### The United States Pharmacopœia.

We have on our table two pamphlats, one proposing and advancing certain changes in the methods of revision and in the character of the United States Pharmacopœia. The first of these is by Dr. Squibb, of New York, who will probably furnish copies to those interested, on making application. The second is by Dr. H. C. Wood, whose pamphlet can be obtained of J. B. Lippincott & Co., of Philadelphia. We shall have more to say of them hereafter.

